# Study Protocol for a Randomized Double Blind, Placebo Controlled Trial Exploring the Effectiveness of a Micronutrient Formula in Improving Symptoms of Anxiety and Depression

**DOI:** 10.3390/medicines5020056

**Published:** 2018-06-14

**Authors:** Meredith Blampied, Caroline Bell, Claire Gilbert, Joseph Boden, Rebecca Nicholls, Julia J. Rucklidge

**Affiliations:** 1 Department of Psychology, University of Canterbury, Christchurch 8140, New Zealand; julia.rucklidge@canterbury.ac.nz; 2 Department of Psychological Medicine, University of Otago, Christchurch 8011, New Zealand; caroline.bell@otago.ac.nz (C.B.); joseph.boden@otago.ac.nz (J.B.); 3 Canterbury District Health Board, Christchurch 8024, New Zealand; claire.gilbert@cdhb.health.nz; 4 Pegasus Health, Primary Health Services, Christchurch 8013, New Zealand; reception@cashmere.pegasus.net.nz

**Keywords:** anxiety, depression, treatment, vitamins, minerals, micronutrients

## Abstract

**Background:** Anxiety and depression are conferring an increasing burden on society. Although treatments exist for both conditions, side effects, and difficulties accessing treatment prevent many people from receiving adequate assistance. Nutritional approaches have demonstrated some success in treating anxiety and depression. We plan to investigate whether a micronutrient formula, Daily Essential Nutrients, improves symptoms of anxiety and depression compared to a placebo in a community recruited sample. **Methods:** This will be a randomized, double blind placebo controlled study (RCT). Two hundred adults will be assigned to either a placebo or micronutrient group (placebo or Daily Essential Nutrients (DEN)) in a 1:1 ratio. Baseline data will be collected for 2 weeks, followed by 10 weeks of placebo or micronutrient intervention. Psychometrics will be used to measure progress and participant safety will be monitored weekly. **Results:** The primary outcome measures will be total scores on three measures of symptom severity at 10 weeks. Linear mixed modelling will be used to measure between group differences and effect sizes will be calculated using pooled mean scores and standard deviations over the course of the trial. **Conclusions:** If effective, micronutrients could provide an alternative treatment, with fewer barriers and adverse events than currently available treatments.

## 1. Introduction

Mental health difficulties are becoming recognized internationally for the burden they place on society and human suffering [1]. Subthreshold depression and limited-symptom panic disorder have been identified as particularly costly in the primary health-care system, with those individuals displaying lower symptom severity often lacking options for further specialist treatment [2,3]. Furthermore, these data do not take into account the more extensive treatment options needed for the many people who meet full criteria for mood or anxiety disorders, with lifetime prevalence ranging from 20–25% in New Zealand [4]. Long-term outcome studies investigating depression and anxiety indicate that individuals suffering from these mental health difficulties access more emergency room care and have higher non-medical costs. They also experience loss of productivity, both from higher work absences due to poor mental health and more indirect damages to productivity due to difficulties managing mental health while at work [5,6]. Depression and anxiety are consistently demonstrated to be common and impairing, at both an individual and societal level.

Best-evidence treatment for anxiety and depression includes both pharmacological approaches and psychological therapies, with a stepped care model generally recommended [7]. However, treatment options have varying response rates for anxiety and depression. 

Pharmacological treatments for anxiety and depression have the benefit of being fairly easily accessible in primary care and often subsidized in many countries [8,9]. Response rates have been well-studied over the past century and provide a range of outcomes from which to inform treatment. Studies have demonstrated small to medium effect sizes comparing a range of psychopharmacological treatments in reducing symptoms of depression and anxiety when compared to placebo [10,11,12,13]. A recent systematic review of 21 antidepressant drugs demonstrated these medications as more effective for treating symptoms of depression than placebo, with odds ratios ranging from 2.13 (amitriptyline) to 1.37 (reboxetine) [14]. However, despite the documented efficacy of medications, side effects including loss of libido, weight gain, physical agitation, and emotional numbness, and symptoms of withdrawal on cessation can be concerning for patients as well as impacting on long-term health and wellbeing and decrease the acceptability of these treatments. [11,15,16,17]. As such, other approaches need to be considered.

Psychological treatments fare better in regards to implementing change without the danger of harmful side effects and have been demonstrated to be efficacious in treating anxiety and depression [18]. However, there are still individuals with psychiatric disorders who do not respond to psychological therapies [19,20,21]. Regardless of psychological treatment offered, some research has indicated that up to 30% of individuals will discontinue therapy, 30–40% will report little or no change, and 5–15% will report negative effects as a result of therapy [22]. In addition, psychological treatments may not be accessible due to lack of availability, service constraints, or cost, even in a stepped care model [6,23].

Poverty of diet has been well identified as problematic for people with a wide range of mental health issues [24,25,26]. Diets of people experiencing mood disorders have been found to be poorer than those without mood disorders [27] and there is a growing body of evidence demonstrating the role of diet and nutrition in improving mental health more generally in adults. A review of randomized controlled trials investigating dietary interventions for depression and anxiety found that although studies struggled with low power and high heterogeneity, there may be positive improvement in depressive symptoms through improving diet [28]. A recent randomized controlled trial examining the impact of a modified Mediterranean diet on depressive symptoms found that those on the modified diet had significantly lower scores on the depression outcome as compared with the social support group, with a large effect size. Participants on the modified diet also improved on other self-report measures of anxiety and depression [29]. Improved nutrient intake has been associated with improved mood in a cross-sectional study that investigated dietary habits in adults with pre-existing mood disorders [30]. Despite this research, making and maintaining dietary changes is complex and often difficult, in particular for individuals struggling with low mood and anxiety [31,32,33,34].

There is considerable interest in nutritional supplementation as a method of circumnavigating the challenges presented in making large-scale, well-maintained dietary changes and subsequently improving mental health outcomes. Specifically, high-dose vitamin B complex has demonstrated success across a range of psychiatric symptoms, including anxiety and depression [35,36]. Improvements in mental health symptoms have also been found for various other single nutrient interventions, including folate [37], omega-3 [38], magnesium [39], and zinc [40]. However, studies on single nutrient interventions have tended to yield small clinical changes or suffered from replication difficulties [41]. Given the complexities of brain function and various epidemiological aspects of mental health, it is possible the single-nutrient interventions lack sufficient heterogeneity to make large scale improvements in mental health and that a broad spectrum, micronutrient approach may be more efficacious [42].

Emerging evidence exploring the impact of broad-spectrum micronutrient supplementation on mental health has found a range of beneficial outcomes. Micronutrients improved depressive symptoms in older adults over six months [43], minimized stress and improved resiliency following various natural disasters in a community sample of adults [44,45] and also in adults with attention-deficit/hyperactivity disorder (ADHD) [46]. Micronutrients have also shown benefit for primary insomnia alongside additional improved mood, stress, and anxiety symptoms in adults [47]. Children with ADHD not only showed better attention and hyperactivity, they also experienced benefits in their mood symptoms and overall functioning when taking micronutrients [48]. Several studies have also demonstrated the effect of micronutrients in improving stress in healthy adults compared to placebo [49,50]. Recent meta-analyses of the literature have indicated that micronutrient treatment has promising implications for improved mental health in pregnancy and child development [51], as well as ameliorating mild psychiatric symptoms, stress, and improved mood in healthy adults [36]. Finally, an extensive review of studies investigating micronutrient interventions for adults experiencing psychiatric conditions, demonstrated effectiveness in treating aggressive behavior, ADHD symptoms, depression, and bipolar disorder [41,52].

Daily essential nutrients (DEN) was selected as the micronutrient formula for this study as it contains a comprehensive range of micronutrients (13 vitamins, 17 minerals, and 4 amino acids) at doses predicted to be sufficient to elicit a treatment response without being likely to elicit adverse effects in the majority of participants.

This paper presents the study protocol for the Nutrients for Mental Health, Anxiety and Depression (NoMAD) trial. This is a randomized controlled trial that aims to explore the effectiveness and safety of micronutrients in alleviating symptoms of anxiety and depression in adults. We predict that participants receiving a micronutrient formula in comparison with placebo will have improved symptoms of anxiety and depression and better overall functioning.

## 2. Materials and Methods

### 2.1. Sample

This study will follow participants for a total of 12 months; 2 weeks of baseline symptom monitoring, 10 weeks of placebo or active intervention (RCT), 10 weeks of active intervention (open label), and a naturalistic follow up completed 12 months after starting baseline. It is a randomized, double blind, parallel-group, placebo controlled trial of a natural micronutrient supplement intervention for individuals experiencing functionally impairing symptoms of anxiety and depression. We will aim to enroll 200 adults from the greater Canterbury region (New Zealand). Recruitment and intervention are anticipated to occur over a two-year time frame. Participants will be randomized to receive either a micronutrient formula, DEN or placebo. A single capsule of DEN contains the micronutrients outlined in Table 1. Participants will take up to 12 pills per day to receive a full dose of the micronutrients.

### 2.2. Study Aims

This study will be the first double-blind (participant and investigators), parallel–group RCT designed to explore the effectiveness and safety of a broad spectrum micronutrient formula compared with placebo in medication-free adults with symptoms of anxiety and depression in the community. We hypothesize that participants given DEN will have improved anxiety and depressive symptoms and better overall mental health functioning than participants given a placebo. Measures of effectiveness will include both standardized psychometrics and additional questions capturing diagnostic criteria for all anxiety and depressive disorders, general levels of anxiety, low mood and stress, alcohol intake, personality traits, suicidality, and other measures of safety such as adverse events. As well as looking at symptom change, we will record the number of treatment responders for both conditions and compare the different rates of response. Participants will be considered treatment responders if they have scored a 1 or 2 on the CGI (very much or much improved), measured at the end of the intervention phase. Cost effectiveness of micronutrient treatment will also be estimated by asking participants about medical treatments accessed and new medications prescribed throughout the trial.

### 2.3. Participant Eligibility

All participants must be between 18 to 65 years of age, must have regular access to the internet and be presenting to their general practitioner (GP) with functionally impairing anxiety and/or depressive symptoms. They also must be considered competent to adhere to a trial protocol, which may include ingestion of up to 12 capsules/day with food. Their written and spoken English must be proficient. Participants will be considered unsuitable for the trial if they have a neurological disorder involving the brain or other central function, other major psychiatric conditions requiring hospitalization, active suicidality, other serious medical conditions, or are known to be allergic to the ingredients of the intervention. Participants will also be ineligible if they are taking medication with central nervous system activation, including psychotropic medications. Participants must have been off these medications for a minimum of four weeks prior to the trial and are not encouraged to cease these medications in order to participate. Pregnant and breastfeeding women will also be excluded from the trial. Participants will be asked to refrain from taking additional dietary supplements for the duration of the trial.

### 2.4. Sample Recruitment

Participants will be recruited through GPs in the greater Canterbury region. Methods of recruiting GPs to refer to the trial include presentations at GP information evenings, presentations at individual GP services, information pamphlets for GPs, and advertising through GP web networks. GPs will assess initial suitability for the trial and will make a referral via the trial website, https://mmp.net.nz/. GPs will discuss appropriate treatment options available for participants, including participation in this study. Participants will be aware of the experimental nature of the trial prior to agreeing to participate.

### 2.5. Study Procedure

#### 2.5.1. Screening Assessment

**Figure 1:**
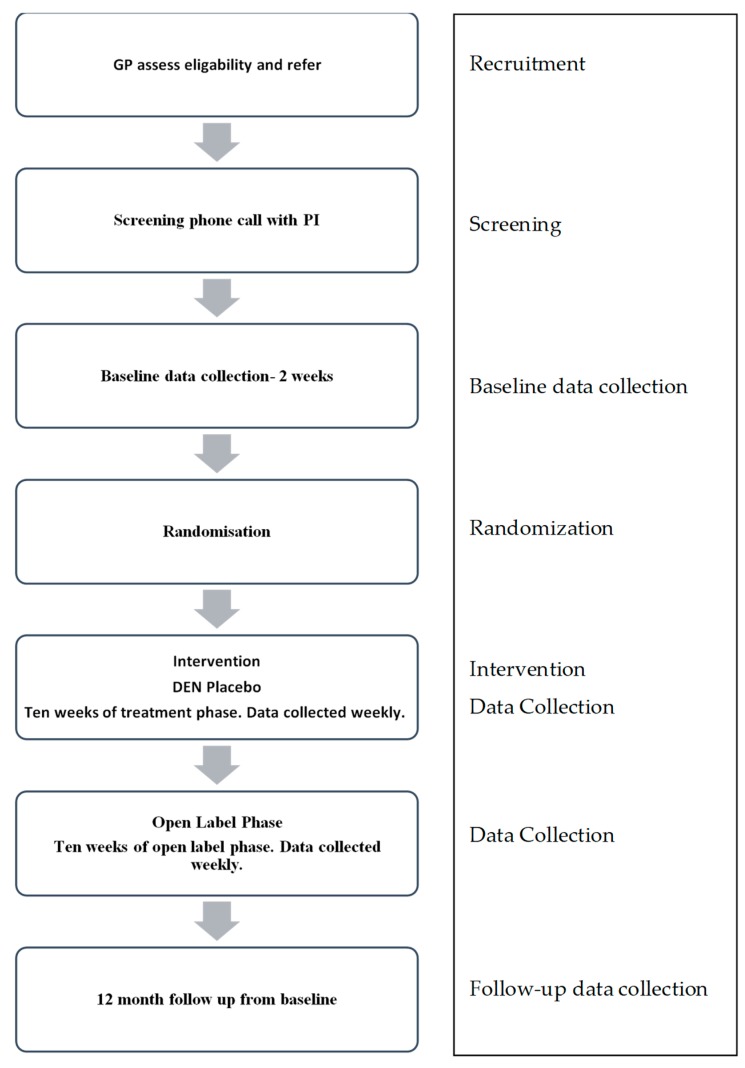
Study process.

Once deemed eligible for the study by their GP, potential participants will be referred directly to the study by the GP using an electronic referral form found on the trial website. The principal investigator (PI) will contact the potential participant once the electronic referral is received. The PI will explain the study, gather informed verbal consent and arrange to collect written consent. If deemed clinically necessary, the PI will offer participants face-to-face interviews at the screening phase. For safety reasons, GPs will be informed if potential participants are accepted or not accepted into the study. If the participant is deemed eligible after this screening, they will be invited to complete remaining baseline data collection questionnaires, which they will access via the trial website, https://mmp.net.nz/. In instances where participants are deemed ineligible for the study, this will be reviewed with the research team and discussed fully with the individual. Appropriate alternative treatment options will be provided to the individual at the time and these treatment options will also be provided to their GP in writing. Any concerns arising during baseline or treatment phase will be managed in the same manner; however, should a participant develop additional difficulties during the treatment phase, they would not be excluded from the study. Should a participant become suicidal during the baseline or treatment phase, immediate information regarding psychiatric crisis support services will be made available to the participant via the trial website. An email will be sent to the PI for follow-up with the participant within 24 h, to assess risk and make immediate recommendations. Changes in participants’ risk status and any recommendations made will also be communicated in writing to the GP. For an overview of the study process, please refer to Figure 1.

#### 2.5.2. Randomization and Allocation

Randomization will be completed by a research assistant who has a purely administrative role in the study that includes no clinical contact with participants, using a program based on the web site randomization.com (http://www.randomization.com) with the randomization sequence arranged in permuted blocks of four. Neither the participants nor the investigators involved in the study will have access to the randomization list, which will be double coded to protect randomization. The pharmacist will be sent the randomization list and will prepare individual participant pill kits in advance, which will contain all required study pills for the 10-week RCT. Information about the intervention and safety related to the invention will be fully explained in an information sheet sent to participants and accessed on the study website. These kits will be sequentially numbered but identical. The intervention kits will be mailed by the research assistant to the individual participants based on the sequential numbering. A sealed envelope containing the treatment allocation for each participant will be kept in a secure location, only to be opened in the case of an emergency (e.g., a serious deterioration in the participant’s health), meaning the blind will be broken for that participant only.

#### 2.5.3. Blinding

Both participants and investigators will be blind to the treatment condition of each participant. There is no difference between the appearance of the placebo or micronutrient product, and both contain riboflavin to ensure the change in urine color this vitamin causes is consistent across the conditions. In addition to riboflavin, the placebo product contains fiber acacia gum, maltodextrin, and cocoa powder. No ingredients include wheat or wheat derivative products and are thus safe for consumption by individuals with gluten allergies. It is noted that the micronutrient product exudes a strong unique smell. Both the micronutrient intervention and placebo will have vanilla sachets added to the pill bottles to conceal any smell that might inform participants of their condition. Once all analyses are complete, the blind will be broken and participants will be informed of their treatment allocation.

#### 2.5.4. Intervention

Participants will begin taking their assigned intervention after a two-week baseline phase. Interventions will be mailed to participants in order to arrive within two days of finishing baseline phase. There will be clear instructions both on the pill bottles (both placebo and intervention) and on the website directing participants to take the intervention only when the baseline phase has completely finished. Participants will initially take one capsule, three times each day. Every second day, the dose will increase by three capsules until a maximum dose of four capsules taken three times a day is achieved: a total dose of 12 capsules per day. Some participants in either condition may need to titrate their dose more slowly and this will be discussed with the participant, the study physician, and the principal investigator. Participants will initially be sent two weeks’ worth of capsules and will then be sent a further four weeks’ worth each month. Participants will continue to take the study interventions for 10 weeks. To measure compliance, participants will be required to send a photo through of their remaining pills weekly and will report the number of pills missed during their weekly questionnaires. Pill compliance photos will be submitted through the website. Questions about adverse events will be asked at each weekly monitoring and participants will have the opportunity to report adverse events at any time during the study, via the trial website or cell phone contact. Should a participant indicate via the trial website that they are experiencing an adverse event, an email will be sent to the PI, who will contact the participant within 24 h of receiving the email. Any reported adverse events will be discussed with the study physician and the research team. In the case of a serious adverse event, the investigators will consider whether termination of the study is necessary. At the completion of the 10 week RCT, participants will repeat psychometric measures given at baseline and all participants will be given the choice to participate in a 10-week open label phase. The open label phase will be completed using the same process as the RCT phase, including re-titrating of capsules. Participants will then be followed up 12 months after the initial baseline phase to complete the same questionnaires as at baseline. The DEN capsules and matching placebo will be donated by Hardy Nutritionals.

### 2.6. Data Collection and Outcome Measures

Table 2 presents all measures and time points at which they are to be collected. Demographic data including previous and current mental health status and treatment expectancies is to be collected at baseline. Participant height and weight will be reported when referred by the GP. Self-report measures will be obtained from each participant at baseline and then weekly during both the treatment and open label phase to measure symptoms of depression and anxiety, overall perceived progress, and any medical interventions required.

#### 2.6.1. Primary Outcome Measure

Participants will complete two psychometrics weekly during the 10-week treatment and 10-week open label phase to measure response to intervention. A clinician-rated measure will provide the third primary outcome measure (CGI-I). The Generalized Anxiety Disorder-7 Question Scale (GAD-7) is a self-report questionnaire that measures the key diagnostic components of Generalized Anxiety Disorder and is commonly used to measure anxiety [53]. Normative data collected from the general population indicates that a score above 10 on the GAD-7 indicates the likely presence of anxiety disorders [53,54]. The Patient Health Questionnaire-9 (PHQ-9) is a self-report tool used widely in community trials to measure depressive symptoms in adults. It has demonstrated good sensitivity and specificity in community samples, both in screening for depression and measuring response to treatment [55,56]. A meta-analysis of the PHQ-9 indicated an appropriate cut-off score of 12 for a diagnosis of major depression in primary care settings; however, did emphasize the need to take into account total sample severity and clinical setting [57].

The PI will complete the Clinical Global Impression-Improvements (CGI-I) at the completion of the treatment and open label phase. The CGI-I measure is widely used in clinical trials to measure subjective change. Clinician ratings using the CGI-I have been demonstrated to be associated with patient-rated and psychometrically measured rates of change [58,59]. Clinicians rate change on a variety of measures, including energy, mood, anxiety, and general functioning. The CGI-I in this study will use a seven-point scale from 1 (very much improved) to 7 (very much worse). At the conclusion of each phase of the study, the PI will review the participants’ outcome measures, take note of comments made during the treatment phases, and their MCGI-I and will make an estimation of improvement using the seven-point scale. The CGI-I global assessment made at the conclusion of the treatment phase will be the primary time-point measured.

#### 2.6.2. Secondary Outcome Measure

Participants will complete these measures up to four times, during the baseline assessment phase, at the completion of the RCT phase, at the completion of the open label phase and the 12-month follow up.

The Modified Clinical Global Impression-Improvement (M CGI-I) draw upon the CGI, which is widely and successfully used in clinical trials to measure subjective change [58,59]. Given the reduced face-to-face contact in this trial, this has been modified for participants to give a subjective rating of their own perceived change on a weekly basis. Participants will be asked to rate how much they thought their mood, anxiety, stress, and energy changed since they started the trial. The M CGI-I will use a seven-point scale, which statement best applied from 1 (very much improved) to 7 (very much worse).

The Depression, Anxiety, and Stress Scale (21 item version) (DASS-21) is a well-validated psychometric that measures core symptoms of anxiety and depression. It has positive psychometric properties and has been used extensively in research settings with community samples to measure changes in symptoms [60].

Developed as a self-report questionnaire from the Brief Social Phobia Rating Scale interview, the Social Phobia Inventory (SPIN) is a 17-item scale designed to measure the presence and severity of social anxiety and to track symptom improvement over time [61]. Adequate psychometric properties were demonstrated in the initial development study and in several further independent studies [62,63,64,65].

Designed to measure the impact of obsessions and compulsions, the Yale-Brown Obsessive Compulsive Scale (Y-BOCS) is a measure that can be administered by clinicians in the form of a semi-structured interview or 10-item self-report scale. The functional impairment self-report measure of the scale will be used to identify the extent and severity of any obsessions and compulsions. A stem question will be used to exclude participants who do not experience obsessions or compulsions. The Y-BOCS self-report scale is widely used in clinical research and has satisfactory psychometric properties [66].

Panic attacks are common occurrences for people experiencing many different mental health issues and can be challenging to differentiate from panic disorder. The Panic Disorder Severity Scale (PDSS) is a seven-item self-report scale that measures the severity of specific panic disorder symptoms, with and without agoraphobia. Research has demonstrated good validity for the self-report measure, although individual items have poor specificity [67,68].

The Impact of Events Scale-Revised (IES-R) is a widely used 22-item self-report measure that assesses subjective distress caused by traumatic events. It is a revised version of the older version, the 15-item IES [69] that now includes measures of hyperarousal that were absent in the original scale. Participants are asked to identify a specific stressful life event and then indicate how much they were distressed or bothered during the past seven days by each “difficulty” listed. Items are rated on a five-point scale ranging from 0 (“not at all”) to 4 (“extremely”). Research on the psychometric properties of the IES-R indicates that it measures the general concept of traumatic stress, best accounted for by a two-factor structure (intrusion/hyperarousal and avoidance). It has high internal consistency and good correlations with other measures of PTSD such as the PTSD checklist (PCL) [70]. A stem question will be used to exclude participants with no exposure to traumatic events.

The Health Anxiety Inventory (HAI) is an 18-item self-report questionnaire designed to measure the cognitive, behavioral, and somatic symptoms of distress about health [71]. Commonly used in clinical and research populations, a recent validation study investigated the psychometric properties of the HAI when administered via the internet [72]. The HAI delivered excellent psychometric properties and cut-off scores have also been validated to indicate severity of health anxiety [72].

The Leeds Dependency Questionnaire (LDQ) is a 10-item self-report questionnaire designed to measure dependence on alcohol and/or opiates [73]. It is flexible in measuring dependence on a wide range of substances and considers both the psychological and physiological aspects of dependence. It has acceptable psychometric properties and has been well validated in adult samples, both in the community and in treatment centers [74,75].

The Quality of Life Scale (QOLS) is 16-item self-report questionnaire that is a reliable and valid instrument for measuring quality of life as rated by the participant [76]. The QOLS measure changes in material wellbeing, physical wellbeing, relationships with people, social activities, community activities, civic activities, personal development, fulfilment, and recreation.

The Antidepressant Side-Effects Checklist (ASEC) and open-ended questions to participants will assess side effects, safety, and adverse events of the intervention [77]. The ASEC has been adapted for the purposes of this study to remove references to antidepressants. Side effects will be assessed weekly during the RCT and open label phase.

#### 2.6.3. Other Measures

The Dietary Screening Tool (DST) is a 25-item self-report questionnaire that assesses dietary intake and identifies those at nutritional risk [78]. Language in the tool has been standardized with New Zealand English e.g., “lollies” instead of “candy”.

Based on Eysenck’s seminal research on personality structure, the Big Five Inventory-10 items (BFI-10) measures personality across five domains-extraversion, agreeableness, conscientiousness, neuroticism, and openness. Recently adapted from a longer, 44-item scale, the BFI 10 has retained adequate psychometric properties when compared to longer, established personality measures [79], in particular demonstrating excellent reliability and validity. Given the number of psychometric tests administered to study participants, the BFI-10 was deemed most suitable for its brevity. This test will only be administered at baseline assessment and will be used to measure any potential personality factors that may be implicated in depression, anxiety and response to micronutrients e.g., neuroticism. At the conclusion of the RCT phase, all participants will also be asked if they thought they were in the treatment or placebo arm.

### 2.7. Data Management

All study data is to be contained in locked storage systems; either a password-protected computer system at the University of Canterbury or on a secure web-based data collection system https://mmp.net.nz/ for electronic documents, while hard copies will be kept in secure filing cabinets at the university.

### 2.8. Study Integrity

The trial has been prospectively registered under the Australian and New Zealand Clinical Trials Registry (ANZCTR). Trial Identification = ACTRN12617001647325. Universal Trial Number (UTN) = U111111994026. Ethics approval was granted through the New Zealand Heath and Disability Ethics Committee 17/STH/131 on 14 November 2017 and the University of Canterbury Human Ethics Committee: HEC2017/108/LR, on 27 November 2017. Written informed consent is to be granted by all participants before entry into this study.

### 2.9. Sample Size

A similar RCT/open label study using the same micronutrient formula provided the basis for the sample size calculations (Protocol for Perinatal Depression & Anxiety RCT 2016, Mental Health and Nutrition Research Group, TRN ACTRN12617000354381). An effect size of d = 0.5 was chosen based on previous studies that examined the treatment of anxiety, depression and stress using multi-micronutrient supplement compared with placebo in adults [45,80,81] and depression outcomes from use of a B vitamin complex compared with placebo on participants with major depressive disorder (d = 0.88) [82]. Assuming an effect size of d = 0.5, the total number of participants required would be 126 (63 in each group). Attrition rates in nutrition RCTs appear to range between 3% to 19% [43,44,45]. The adjusted number per treatment condition would be 75 and the total sample size therefore would be 150. However, general treatment RCTs have demonstrated attrition rates ranging between 0.8% and 48%, with varying degrees of attrition linked to differences in trial design and outcome e.g., face-to-face assessment or treatment, web-based treatment, severity of adverse events [83,84,85]. Given the reduced face-to-face follow up, this study will allow for an anticipated 50% attrition rate. However, to randomize in blocks of four and to allow for excess attrition given the reduced face-to-face contact during the trial, the study will aim to recruit 200 participants, 100 in the treatment arm and 100 in the control arm. It is noted that sufficient power will be attained once 126 participants have been recruited.

### 2.10. Data Analyses

This study aims to enroll participants from June 2018. Final data collection is anticipated to be completed in June 2020.

Descriptive statistics including age, socioeconomic status, and occupation will be documented in order to compare the two groups’ homogeneity to each other and the wider population. Two-tailed *t*-tests or ANOVA will be used to identify any group differences.

The intention to treat (ITT) population for the primary analyses of outcomes will include all randomized participants regardless of whether they complied with capsule consumption. Treatment adherence will be based on total treatment consumption and using a per-protocol analysis (PP). The (PP) population for each primary and secondary measure will include all participants who take at least 80% of the allocated pills, have no significant protocol deviations and have all appropriate assessments relevant to the outcome. The safety population, which will be used for all safety analyses, will include all participants who have taken at least one dose of study product. Treatment groups will be defined as the actual treatment received irrespective of randomized treatment.

For the primary outcomes (GAD-7 and PHQ-9), the repeated measures of the outcome variables will be modelled using generalized linear mixed effects regression models. These models will permit the testing of differences between the micronutrient group and the placebo group over the course of the trial. Baseline scores on the primary outcome measures will be used as covariate factors, as well as measures of demographic characteristics. The pooled mean scores (and standard deviations) over the course of the trial on each of the primary outcomes will be used to compute estimates of effect size (Cohen’s d). For the CGI-I, the groups will be compared at the end of study treatment using *t*-tests as the CGI-I will be made only at the conclusion of the 10-week intervention period. The CGI-I will also be reported as responder/non-responder by group using chi-square analyses/odds ratios. A score of 1 or 2 (very much improved and much improved) will be used to identify responders.

For secondary outcomes, linear mixed effects models will also be used. For data from randomized trials, this modelling procedure allows the researcher to fit individual-specific slopes and intercept terms, which can account for individual variability in treatment response more precisely than methods based on Analysis of Variance. The statistical test for differences between groups will be an F test. For secondary outcomes, linear mixed effects models will also be used, to explore the relationship between other psychiatric difficulties and the micronutrient intervention.

Finally, modified Brinley Plots [47,86,87] will be constructed to show overall change, along with Reliable Change for each individual, overall % Reliable Positive Change, and Cohen’s d Effect Size. pre- and post-treatment data points for each participant will be placed onto a scatter plot, where the direction of desired change and clinical cut-offs are indicated to assist interpretation.

Treatment-emergent adverse events and risk events from the treatment phase will be individually listed by randomized group, indicating the preferred term, date of onset, date resolved, severity, relatedness, frequency, action taken in relation to study medication, and whether the adverse event is serious. The incidence of more common adverse events (greater than 5%) or adverse events of special interest may also be summarized for each randomized group.

## 3. Discussion

As depression and anxiety continue to place increased burden on individuals, healthcare systems, and society more broadly, the need is urgent to identify effective treatment options for these conditions. Currently recommended treatment options are available and have a range of effective outcomes [21,88]. However, continued blocks to these treatments persist, including cost, side-effects and non-responders [23,89]. Although dietary and single nutrient studies have demonstrated some efficacy in reducing symptoms of anxiety and depression, these have high heterogeneity and difficulties in replication [28,41]. The current study aims to assess the effectiveness of a broad spectrum micronutrient formula compared to a placebo in improving symptoms of anxiety and depression. We will also explore effects of micronutrient intervention on a range of other life factors, including quality of life and substance use, and continue to assess the safety of the micronutrient intervention. Cost effectiveness of a micronutrient intervention will also be explored, given the high prevalence of anxiety and depressive symptoms in the general population. This is a protocol paper outlining a future randomized controlled trial. While publishing protocols prospectively is encouraged as a method of promoting ethical data collection, it will also be important to publish the data from the trial for scientific review.

In regards to limitations of the trial, the authors acknowledge that nutrient intake via detailed dietary records and/or nutrient intake via blood serum is not being measured. While it may be desirable to measure more variables, given that this study is the first to empirically test the efficacy of micronutrients on anxiety and depression in a community setting, it provides a unique contribution to the current literature without this information. Indeed, blood nutrient biomarkers do not always correlate well with psychological measures [82] and therefore at this stage cannot be used reliably to identify those who may be deficient in specific nutrients. This proposed randomized controlled trial serves as an initial rigorous test of the efficacy of broad spectrum micronutrient supplementation and the randomization process ensures that both groups should be matched across many different variables. Should micronutrients be deemed efficacious, further studies could and should explore targeted personalized approaches to supplementation, gathering detailed nutrient information, from both diet records and biomarkers, and use those to determine optimal treatment. Both dietary intervention and supplementation studies using many different designs are necessary to inform on the merits of improving mental health with nutrition.

## Figures and Tables

**Table 1:** Nutrient information

**Daily Essential Nutrients (DEN) Advanced Supplement Facts**			
Amount Per Full Dose (12 capsules)			
Vitamin A (as retinyl palmitate)	5760 IU	Calcium (as NutraTek™ chelation complex)	1320 mg
Vitamin C (as ascorbic acid)	600 mg	Iron (as NutraTek™ chelation complex)	13.8 mg
Vitamin D (as cholecalciferol)	3000 IU	Phosphorus (as NutraTek™ chelation complex)	840 mg
Vitamin E (as d-alpha tocopheryl succinate)	360 IU	Iodine (as NutraTek™ chelation complex)	204 mcg
Thiamin (as thiamin mononitrate)	60 mg	Magnesium (as NutraTek™ chelation complex)	600 mg
Riboflavin	18 mg	Zinc (as NutraTek™ chelation complex)	48 mg
Niacin (as niacinamide)	90 mg	Selenium (as NutraTek™ chelation complex)	204 mcg
Vitamin B6 (as pyridoxine hydrochloride)	69.9 mg	Copper (as NutraTek™ chelation complex)	7.2 mg
Folate (as calcium l-5 methyletrahydrofolate)	828 mcg	Manganese (as NutraTek™ chelation complex)	9.6 mg
Vitamin B12 (as methylcobalamin & adenosylcobalamin)	900 mcg	Chromium (as NutraTek™ chelation complex)	624 mcg
Biotin	1080 mcg	Molybdenum (as NutraTek™ chelation complex)	144 mcg
Pantothenic acid (as d-calcium pantothenate)	30 mg	Potassium (as NutraTek™ chelation complex)	240 mg
Propriety blend: Choline bitartrate, alpha-lipoic acid, mineral wax, inositol, acetyl-l carnitine, grape seed extract, gingko biloba leaf extract, *N*-acetyl-l-cysteine, l-methionine, trace minerals as NutraTek™ chelation complex, lithium orotate, boron, vanadium, nickel.Other ingredients: Vegetarian capsule (hypromellose), microcrystalline cellulose, magnesium stearate, silicon dioxide, titanium dioxide.			
**Placebo Facts**			
Amount Per Full Dose (12 capsules)			
Fiber Acacia Gum	3600 mg	Cocoa Powder	48 mg
Maltodextrin	4750.8 mg	Riboflavin Powder	1.2 mg

**Table 2:** Schedule of measurements

Variable	Instrument	Time Point
Self-report		
Primary outcome measures (anxiety and depressive symptoms)	Generalised Anxiety Disorder-7 Question Scale (GAD-7) Patient Health Questionnaire-9 (PHQ-9)	Baseline, weekly during treatment, end of treatment, weekly during open label, end of open label, one-year follow up.
Anxiety symptoms	Social Phobia Inventory (SPIN) Yale-Brown Obsessive Compulsive Scale (Y-BOCS) Panic Disorder Severity Scale (PDSS) Impact of Events Scale-Revised (IES-R) Health Anxiety Inventory (HAI)	Baseline, end of treatment, end of open label, one year follow up.
Psychiatric status	Web Screening Questionnaire (WSQ) Depression, Anxiety and Stress Scale (DASS-21)	Baseline, end of treatment, end of open label, one-year follow up.
Personality	Big Five Inventory (BFI-10)	Baseline
Quality of life	Quality of Life Scale (QOLS)	Baseline, end of treatment, end of open label, one-year follow up.
Diet quality, alcohol and drug intake	Dietary Screening Tool (DST), Leeds Dependency Questionnaire (LDS)	Baseline, end of treatment, end of open label, one-year follow up.
Treatment side effects	Modified Antidepressant Side-Effects Checklist (ASEC)	Baseline, weekly during treatment, end of treatment, weekly during open label, end of open label.
Treatment effectiveness	Modified Clinical Global Impression-Improvements (MCGI-I)	End of treatment, end of open label, one year follow up.
Observer report		
Treatment effectiveness	Clinical Global Impression-Improvement (CGI-I) *	End of treatment, end of open label, one year follow up.

* Primary outcome measure.
